# School age outcomes of very premature infants randomized to cord milking versus early cord clamping at birth

**DOI:** 10.1007/s00431-025-06299-y

**Published:** 2025-07-01

**Authors:** Kamile Akyol Ozkara, Serdar Alan, Ezgi Ozalp Akin, Emel Okulu, Emine Bahar Bingoler Pekcici, Elcin Caglar, Omer Erdeve, Begüm Atasay, Saadet Arsan

**Affiliations:** 1 Department of Pediatrics, Ankara University School of Medicine, Ankara, Turkey; 2 Division of Neonatology, Department of Pediatrics, Kirikkale University School of Medicine, Yahsihan/Kırıkkale, Turkey; 3 Division of Developmental Behavioral Pediatrics, Department of Pediatrics, Ankara University School of Medicine, Ankara, Turkey; 4 Division of Neonatology, Department of Pediatrics, Ankara University School of Medicine, Ankara, Turkey

**Keywords:** Umbilical cord milking, Placental transfusion, Very preterm newborn, Long term outcome, Wechsler intelligence scale for children, Vineland adaptive behaviour scales

## Abstract

To evaluate the effect of intact cord milking (I-UCM) compared to immediate cord clamping (ICC) on neurodevelopmental outcomes at seven years of age in very preterm infants. This prospective single-blind cohort study included children who were previously participated in a randomized controlled trial comparing I-UCM and ICC. At about 7 years of age, participants were administered the Vineland Adaptive Behavior Scales, Second Edition (Vineland-II) and the Wechsler Intelligence Scale for Children, Fourth Edition (WISC-IV). A total of 31 children were included in the follow-up study. The mean age of participants was 6.4 ± 0.5 years. The mean gestational age at birth was 28.5 ± 1.7 weeks. There were no cases with grade ≥ 3 intraventriculary hemorrhage (IVH) in the present study cohort. Although the I-UCM group showed a trend toward higher median full-scale IQ, the difference was not statistically significant (*p*: 0.057). A significantly higher percentage of cases in the I-UCM group achived a full-scale IQ above 85 in the WISC-IV (*p* = 0.048). The mean of the "written language scaled score" subdomain among Vineland-II scores was found to be significantly higher in the I-UCM group. A significantly higher percentage of cases in the I-UCM group had a written language scaled score above 12 (*p*: 0.029).

*Conclusion*: A comparison of I-UCM with ICC in preterm infants born at a mean age of 28 weeks and without severe IVH revealed that I-UCM did not result in long-term neurodevelopmental adverse outcomes. I-UCM even had positive effects in some subdomains of detailed neurodevelopmental tests.

**Table:** 

**What is Known:** • *Compared to immediate cord clamping, umbilical cord milking improves the short-term postnatal outcomes of very preterm infants. There is a lack of robust data regarding the long-term neurodevelopmental effects of umbilical cord milking in preterm infants.*
**What is New:** • *Among very preterm infants without severe intraventricular hemorrhage, intact umbilical cord milking was not associated with long-term adverse neurodevelopmental outcomes. Intact-umbilical cord milking even had positive effects in some subdomains of detailed neurodevelopmental tests.*

**What is Known:**

• *Compared to immediate cord clamping, umbilical cord milking improves the short-term postnatal outcomes of very preterm infants. There is a lack of robust data regarding the long-term neurodevelopmental effects of umbilical cord milking in preterm infants.*

**What is New:**

• *Among very preterm infants without severe intraventricular hemorrhage, intact umbilical cord milking was not associated with long-term adverse neurodevelopmental outcomes. Intact-umbilical cord milking even had positive effects in some subdomains of detailed neurodevelopmental tests.*

## Introduction

Placental transfusion refers to the transfer of residual blood from the placenta to the newborn between birth and umbilical cord clamping [1]. Two main strategies, deferred/delayed cord clamping (DCC) and umbilical cord milking/stripping (UCM), are used to achieve placental transfusion [2]. DCC allows for a smoother hemodynamic transition at birth by improving pulmonary blood flow compared with immediate cord clamping (ICC) [2–4]. Increasing evidence supports the benefits of DCC over ICC for both preterm and term infants, with improvements noted in both short- and long-term outcomes [5]. Furthermore, DCC is associated with improved motor function in preterm infants at 12–18 months [6, 7].

Intact umbilical cord milking (I-UCM) is defined as grasping the unclamped umbilical cord blood and milking it toward the neonate 2 to 4 times before the cord is clamped [1]. This method effectively increases blood volume, hemoglobin, and hematocrit levels in preterm infants born after the 28 weeks of gestation without resulting in serious adverse events [4]. UCM was used as an alternative method to DCC for placental transfusion until the study comparing the effects of DCC and UCM on intraventricular hemorrhage (IVH) and death in preterm infants born under 28 weeks’ gestation was published in 2019 by Katheria et al. [8]. They had to stop the study because they found that I-UCM increased the risk of intraventricular hemorrhage by a significant amount in preterm infants under 28 weeks compared to DCC [8]. In animal studies, it has been suggested that UCM may lead to IVH by causing significant carotid artery fluctuation in preterm infants, who already have an impaired ability to regulate cerebral circulation due to immaturity, if UCM is performed before the onset of breathing [9, 10]. These fluctuations may be mitigated by ensuring that UCM is conducted after the initiation of spontaneous breathing and allowing cord refill from the placental side [9, 10]. On the other hand, no evidence has been found that UCM increases the rates of IVH in preterm infants when compared to ICC [11]. This would suggest that that UCM remains a reasonable option when DCC is not feasible.

Despite its frequent use, there remains a lack of robust data regarding the long-term neurodevelopmental effects of UCM in preterm infants [12, 13]. To our knowledge, only one study has specifically compared the long-term neurodevelopmental outcomes of preterm infants receiving UCM versus ICC [11]. Given the knowledge gap, further studies are required to evaluate the long-term safety and efficacy of UCM, particularly in very preterm infants.

This study aimed to assess the impact of I-UCM compared to ICC on neurodevelopmental outcomes at 7 years of age in very preterm infants.

## Material and methods

### Study design and participants

This study reports the long-term follow-up findings of a previously published randomized controlled trial (RCT) conducted at a tertiary perinatal center between April 2011 and February 2013. The original study compared the short-term effects of UCM versus ICC in very preterm infants [14]. All participants were analyzed according to the groups to which they were originally randomized, in accordance with the intention-to-treat principle.

Eligible infants had a gestational age < 32 weeks and an estimated birth weight < 1500 g, as assessed by the obstetrics team. Exclusion criteria included: (a) suspected twin-to-twin transfusion syndrome or discordant twins; (b) major congenital or chromosomal anomalies; (c) maternal vaginal bleeding due to placenta previa, placental abruption, or placental tear; (d) hemolytic disease of the fetus and newborn (e.g. Rh isoimmunization); (e) intrauterine growth restriction; (f) maternal gestational diabetes requiring insulin; (g) hydrops fetalis; and (h) lack of parental consent [14].

Randomization was performed immediately prior to delivery using sequentially numbered, non-transparent envelopes. In twin pregnancies, the first twin was randomized, and the second was assigned to the opposite intervention group. The attending neonatologist informed the obstetrician of the assigned intervention [14]. In the I-UCM group, the neonate was held at the level of the placenta during cesarean section (CS) delivery and below the placenta during vaginal delivery. Approximately 25–30 cm of the cord was milked toward the infant three times, allowing placental refill between each milking (performed by: S.A.). The onset of spontaneous breathing during UCM was not recorded. The milking speed was approximately 5 cm/second. In the ICC group, the umbilical cord was clamped within 10 s of birth [14].

The primary outcome of the original study was the number and volume of red blood cell (RBC) transfusions within the first 35 days of life. Secondary outcomes included hemodynamic parameters during the first 24 h. Demographic and clinical data, including preterm morbidities, were collected from the hospital records. Based on a projected 20% reduction in RBC transfusion rates, a sample size of 19 infants per group was calculated to 90% power and an alpha of 0.05 [14].

### Follow-up study protocol

The current single-blind cohort study was conducted between May 2018 and February 2019, when the study participants were between 70 and 89 months of age. This study was performed in line with the principles of the Declaration of Helsinki. Ethical approval was granted by the Ethics Committee of Ankara University Faculty of Medicine (Approval date: 14.08.2017, No: 13–830-17).

Parents of the 38 surviving children from the original trial were contacted by a researcher (K.A.O.). Children whose parents provided written informed consent were enrolled. Participants underwent comprehensive assessments including neurological examination, cognitive and adaptive development evaluation, and hearing and vision testing. The researchers (E.O.K. and E.B.B.P.) conducting the neurodevelopmental assessments were blinded to the children’s original group allocation.

### Procedures

Vineland Adaptive Behaviour Scales, Second Edition (Vineland-II): The Vineland-II was used to assess adaptive functioning, defined as age-appropriate skills necessary for personal and social independence. These skills are performed independently in daily routines to cope with environmental demands [15]. The Vineland-II includes four domains and related subdomains: Communication (receptive, expressive and written language); Daily Living Skills (personal, community and domestic); Socialization (interpersonal relationships, play and coping), and Motor skills (fine and gross motor) [16]**.** Domain scores are combined to yield an “adaptive behaviour composite (total) score”. Subdomain scaled scores have a mean of 15 and a standard deviation of 3, and composite scores have a mean of 100 and a standard deviation of 15. An adaptive behaviour composite score below 70 indicates significant developmental delay (> 2 standard deviation below mean). A score below 85 reflects mild developmental delay (> 1 standard deviation below mean). Each interview was conducted by a blinded developmental pediatrician during a 60–90-min semi-structured interview with the child’s parent.

Wechsler Intelligence Scale for Children, Fourth Edition (WISC-IV): The WISC-IV is a widely used tool to assess intelligence in children aged 6–16 years. It includes 10 core subtests used to calculate four index scores: Verbal Comprehension Index, Perceptual Reasoning Index, Working Memory Index, and Processing Speed Index. These indexes combine to form the “full-scale IQ, with a mean of 100 and a standard deviation of 15. A full-scale IQ below 85 indicates mild cognitive delay, and below 70 indicates significant delay [17].

Hearing Assessment: Brainstem Auditory Evoked Response (BAER) testing was used to assess the auditory function. Results were interpreted by a pediatric neurologist blinded to group allocation.

Ophthalmologic Assessment: A pediatric ophthalmologist, also blinded to group assignment, evaluated visual acuity (monocular and binocular), color vision, fusion, stereopsis, and light reflex. Comphrensive eye exams included strabismus evaluation, biomicroscopy and retinoscopy.

### Statistical analysis

Descriptive statistics were presented as frequencies and percentages for categorical variables, and as means ± standard deviations or medians with interquartile ranges for continuous variables, depending on the distribution. Normality of data was assessed using appropriate statistical tests. Comparisons between the I-UCM and ICC groups were conducted as follows: Student’s t-test was used for normally distributed continuous variables; Mann–Whitney U-test was used for non-normally distributed continuous variables; Chi-square or Fisher’s exact test was used for categorical variables In addition, biivariate analyses were performed to explore the association between socioeconomic variables (maternal education and household income) and neurodevelopmental outcomes, particularly adaptive behaviour and full-scale IQ scores ≤ 85. A p < 0.05 was considered statistically significant. Statistical analyses were performed using the IBM SPSS 20.0 (SPSS Inc., Chicago, IL, USA).

## Results

Of the 44 infants originally enrolled in the RCT, 4 (2 from each group) died during the neonatal period. Both infants in the UCM group who died had grade ≥ 3 IVH. Among the 40 surviving infants, 4 were lost to follow-up, and 5 families declined participation. One of the infants lost to follow-up also had grade ≥ 3 IVH and belonged to the UCM group.

**Fig. 1:**
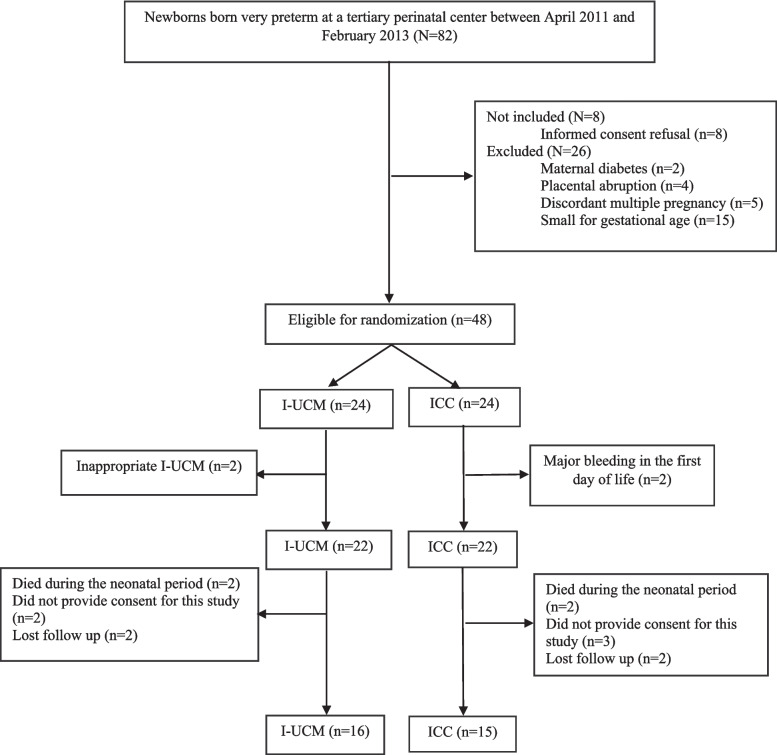
Flowchart of the research and inclusion/exclusion of cases

A total of 31 children (77.5% follow-up rate) were included in this study: 16 in the I-UCM group and 15 in the ICC group (Fig. 1). The mean age at follow-up was 6.4 ± 0.5 years, and 51.6% (*n* = 16) were male. The mean gestational age was 28.5 ± 1.7 weeks, and 77.4% (*n* = 24) were delivered via CS.

The sociodemographic and clinical characteristics of the cases are summarized in Table 1. The I-UCM and ICC groups were comparable in terms of age, sex, birth weight, household income, and neonatal morbidities, including the need for resuscitation at birth, respiratory distress syndrome, early- and late-onset neonatal sepsis, patent ductus arteriosus, necrotizing enterocolitis, and grade ≤ 2 IVH.

**Table 1:** A comparison of the sociodemographic and clinical characteristics of the I-UCM and ICC groups (*n* = 31)

	I-UCM (*n*: 16)	ICC (*n*: 15)	*p*
Current age (years, mean ± SD)	6.53 ± 0.28	6.35 ± 0.67	0.357
GA (weeks, mean ± SD)	28.68 ± 1.59	28.33 ± 1.87	0.572
Male (%)	56.3	46.7	0.594
Female (%)	43.8	53.3	
**Morbidities related to prematurity**			
The need for resuscitation at birth (%)	43.8	26.7	0,320
Respiratory distress syndrome (%)	68.8	40.0	0.108
Early onset neonatal sepsis (%)	62.5	66.7	0.809
Late onset neonatal sepsis (%)	81.3	80.0	0.641
Patent ductus arteriosus (%)	12.5	20.0	0.654
Necrotizing enterocolitis (%)	0.0	6.7	0.484
Intraventricular hemorrhage (%)	0.0	6.7	0.484
**Family characteristics**			
Maternal education			
Primary school (%)	33.3	33.3	
High school graduate (%)	43.7	33.3	0.896
University education or higher (%)	25.0	33.3	
Household income ≤ minimum wage (%)	18.8	26.7	0.685
Multiple children (%)	75.0	66.7	0.704

*I-UCM* Intact umbilical cord milking, *ICC *Immediate cord clamping

Socioeconomic influence on development is summarized in Table 2. No significant association was found between maternal education level and their children’s long-term neurodevelopmental scores. However, children from households with income above the minimum wage demonstrated significantly higher scores in several domains including adaptive behavioral composite score, communication domain score, socialization score, motor domain standard score, and WISC-4 processing speed index (*p* = 0.009, 0.01, 0.048, 0.034, and 0.04, respectively) than incomes equal to or less than minimum wage.

**Table 2:** The relationship between cognitive scores and maternal education and income level

**Maternal education**	**Before high-school** **(*n*: 10)**	**Equal or higher than high-school** **(*n*: 21)**	*p*
**Vineland-II scores**			
Adaptive behavior composite (total)	84.1 ± 11.1	82.9 ± 9.2	0.754
Communication domain standard	85.7 ± 13.3	89.1 ± 15.5	0.551
Daily living skills standard	86.4 ± 8.1	86.4 ± 7.2	0.992
Socialization domain standard	86.8 ± 11.3	84.9 ± 8.2	0.609
Motor skills domain standard	81.3 ± 11.6	79.2 ± 7.5	0.612
**WISC-IV scores**			
Verbal comprehension index	92.0 ± 12.9	99.0 ± 12.3	0.159
Perceptual reasoning index	81.8 ± 18.0	86.8 ± 11.8	0.356
Working memory index	80.8 ± 20.2	87.2 ± 16.3	0.350
Processing speed index	87.8 ± 20.9	95.0 ± 13.0	0.251
**Monthly income**	**Lower than minimum wage** **(*n*: 7)**	**Higher than minimum wage** (*n*: 24)	***p***
**Vineland-II scores**			
Adaptive behavior composite (total)	75.1 ± 7.6	85.7 ± 9.0	**0.009**
Communication domain standard	75.9 ± 8.6	91.6 ± 14.3	**0.010**
Daily living skills standard	82.0 ± 6.7	87.7 ± 7.2	0.071
Socialization domain standard	79.6 ± 8.9	87.3 ± 8.6	**0.048**
Motor skills domain standard	72.8 ± 8.8	81.8 ± 7.5	**0.034**
**WISC-IV scores**			
Verbal comprehension index	90.9 ± 15.7	98.4 ± 11.6	0.171
Perceptual reasoning index	77.7 ± 16.9	87.4 ± 12.6	0.108
Working memory index	79.7 ± 23.8	86.8 ± 15.6	0.361
Processing speed index	77.9 ± 15.7	97.0 ± 13.5	**0.004**

The data are presented as the mean ± SD

*Vineland-II *Vineland Adaptive Behavior Scales Second Edition, *WISC-IV* Weschler Intelligence Scale for Children Fourth Edition

Table 3 presents a comparison of the I-UCM and ICC groups in terms of Vineland-II and WISC-4 scores. The I-UCM group had a higher median IQ than the ICC group: 93 (40–106) versus 83 (57–104), respectively (p: 0.057). Mean adaptive behavior composite scores were 84.9 ± 11.9 in the I-UCM group and 81.5 ± 6.5 in the ICC group (*p*: 0.340).

**Table 3:** Comparison of Vineland-II and WISC-IV scores between I-UCM and ICC groups

	I-UCM group (*n*: 16)	ICC group (*n*: 15)	*p*
**Vineland-II Scores**			
Adaptive behavior composite (total)	84.9 ± 11.9	81.5 ± 6.5	0.336
Communication domain standard	91.4 ± 18.4	84.4 ± 8.5	0.187
a. Receptive language scaled score	13.3 ± 2.8	13.9 ± 1.8	0.469
b. Expressive language scaled score	13.5 ± 2.1	12.9 ± 1.9	0.377
c. Written language scaled score	13.3 ± 3.3	10.6 ± 3.4	**0.036**
Daily living skills standard scores	88.1 ± 8.4	84.7 ± 5.9	0.206
a. Personal daily living skills scaled	12.5 ± 2.0	12.1 ± 1.6	0.575
b. Community daily living skills scaled	13.7 ± 1.5	13.3 ± 1.4	0.431
c. Domestic daily living skills scaled	13.3 ± 1.3	12.4 ± 1.1	0.066
Socialization domain standard scores	86.4 ± 10.6	84.6 ± 7.6	0.586
a. Interpersonal relationships scaled	12.7 ± 1.5	12.1 ± 1.2	0.264
b. Play and leisure time scaled score	13.3 ± 2.5	13.6 ± 1.9	0.723
c. Coping skills scaled score	11.8 ± 2.5	11.1 ± 1.9	0.413
Motor skills domain standard score	78.8 ± 9.6	80.9 ± 7.5	0.584
a. Gross motor scaled score	11.9 ± 2.4	12.3 ± 2.3	0.705
b. Fine motor scaled score	11.3 ± 1.4	11.5 ± 1.3	0.671
**WISC-IV scores**			
1. Verbal comprehension index	100.2 ± 13.3	92.9 ± 11.4	0.111
2. Perceptual reasoning index	87.7 ± 15.1	82.6 ± 12.7	0.320
3. Working memory index	90.9 ± 16.4	79.0 ± 17.2	0.058
4. Processing speed index	93.4 ± 17.7	91.9 ± 14.5	0.798

The data are presented as the mean ± SD

*Vineland-II* Vineland Adaptive Behavior Scales Second Edition,* WISC-IV* Weschler Intelligence Scale for Children Fourth Edition, *I-UCM* Intact umbilical cord milking, *ICC* Immediate cord clamping

In the domain-level analysis, a significantly higher percentage of children in the I-UCM group achieved a written language scale score greater than 12 compared to those in the ICC group (*p*: 0.029). Conversely, the receptive language domain showed an unexpected pattern, with a significantly higher proportion of children in the ICC group attaining scaled scores above 12 compared to the I-UCM group (*p*: 0.023) (Table 4). Additionally, a significant greater percentage of children in the I-UCM group scored above 85 on the full-scale IQ (*p*: 0.048) (Table 5).

**Table 4:** Comparison of mild developmental delays according to Vineland-II scores between the I-UCM and ICC groups

Vineland-II Scores	I-UCM group (*n*: 16)	ICC group (*n*: 15)	*p*
Adaptive behavior composite (total) score			
≤ 85	47.4	52.6	0.552
> 85	58.3	41.7	
Communication domain standard score			
≤ 85	35.3	64.7	0.073
> 85	71.4	28.6	
1. Receptive language scaled score			
≤ 12	81.8	18.2	**0.023**
> 12	35.0	65.0	
2.Expressive language scaled score			
≤ 12	40.0	60.0	0.458
> 12	57.1	42.9	
3.Written language scaled score			
≤ 12	33.3	66.7	**0.029**
> 12	76.9	23.1	
Daily living skills standard score			
≤ 85	45.0	55.0	0.320
> 85	63.6	36.4	
1.Personal daily living skills scaled score			
≤ 12	52.6	47.4	0.886
> 12	50.0	50.0	
2.Domestic daily living skills scaled score			
≤ 12	42.9	57.1	0.685
> 12	54.2	45.8	
3.Community daily living skills scaled score			
≤ 12	30.8	69.2	0.073
> 12	66.7	33.3	
Socialization domain standard score			
≤ 85	55.0	45.0	0.611
> 85	45.5	54.5	
1.Interpersonal relationships scaled score			
≤ 12	43.8	56.2	0.366
> 12	60.0	40.0	
2.Play and leisure time scaled score			
≤ 12	54.5	45.5	0.809
> 12	50.0	50.0	
3.Coping skills scaled score			
≤ 12	47.8	52.2	0.685
> 12	62.5	37.5	
Motor skills domain standard score			
≤ 85	62.5	37.5	0.348
> 85	33.3	66.7	
1.Gross motor scaled score			
≤ 12	53.3	46.7	1.000
> 12	57.1	42.9	
2.Fine motor scaled score			
≤ 12	52.6	47.4	1.000
> 12	66.7	33.3	

The data are presented as the percentage (%)

*Vineland-II* Vineland Adaptive Behavior Scales Second Edition, *I-UCM* Intact umbilical cord milking, *ICC* Immediate cord clamping

**Table 5:** Comparison of mild developmental delay according to WISC-IV scores between the IUCM and ICC groups

WISC-IV Score	I-UCM (*n*: 16)	ICC (*n*: 15)	*P*
Verbal comprehension index			
≤ 85	25.0	75.0	0.333
> 85	55.6	44.4	
Perceptual reasoning index			
≤ 85	44.4	55.6	0.347
> 85	61.5	38.5	
Working memory index			
≤ 85	37.5	62.5	0.104
> 85	66.7	33.3	
Processing speed index			
≤ 85	44.4	55.6	0.704
> 85	54.5	45.5	
Full Scale IQ			
≤ 85	30.8	69.2	**0.048**
> 85	66.7	33.3	

The data are presented as the percentage (%)

*WISC-IV* Weschler Intelligence Scale for Children Fourth Edition, *ICC* Immediate cord clamping, *I-UCM* Intact umbilical cord milking

Regarding visual assessments, children in the I-UCM group demonstrated superior outcomes across all measures. The median monocular visual acuity in the right eye was significantly higher in the I-UCM group [1.0 (IQR: 0.8–1.0)] compared to the ICC group [0.7 (IQR: 0.5–1.0; *p*: 0.003]. Similarly, the left eye visual acuity was also better in the I-UCM group [1.0 (IQR: 0.6–1.0) vs. 0.8 (IQR: 0.5–1.0); *p* = 0.01]. Binocular visual acuity was also significantly higher in the I-UCM group [1.0 (IQR: 0.8–1.0)] compared to the ICC group [0.8 (IQR: 0.6–1.0); *p*: 0.01].

Regarding hearing outcomes, only one child in the cohort required a hearing device due to sensorineural hearing loss. All the other children (*n* = 30) had a normal BAER test results.

## Discussion

To our knowledge, no previous study in the literature has compared very long-term (70–89 months of age) neurodevelopmental outcomes between I-UCM and ICC groups. Because our children were older, we were able to administer more detailed and advanced neurodevelopmental tests, such as the Vineland-II and the WISC-IV. We were able to evaluate the neurodevelopment of the cases more detailed than in previous studies. According to results of the presents study, the ICC group had a higher percentage of cases involving mild delays in some domains of neurodevelopmental tests.

In this long-term follow-up study, a significantly higher percentage of children in the I-UCM group had a full-scale IQ greater than 85 on the WISC-IV compared to the ICC group. While the I-UCM group also demonstrated a trend toward higher median full-scale IQ, the difference did not reach statistical significance. Improtantly, Vineland-II results showed that the mean written language scaled score was significantly higher in the I-UCM group, with a greater proportion of children scoring above 12 in this subdomain. These findings suggest that I-UCM may offer subtle but meaningful advantages in cognitive and language development in very preterm infants. In contrast, a higher percentage of children in the ICC group scored above 12 in the receptive language subdomain, an unexpected result that may warrant further exploration. This discrepancy could potentially reflect unmeasured postnatal environmental influences such as language exposure or early interventions, rather than a direct effect of cord management strategy. Nevertheless, taken together, the cognitive and adaptive function outcomes favor the I-UCM group.

In a RCT evaluating the effect of DCC versus ICC on IVH and 18-month motor outcomes in preterm infants, it was found that placental transfusion (DCC with one time UCM or 2 to 3 times UCM) protects against motor scores below 85 on the Bayley Scales of Infant and Toddler Development III (Bayley-III) at the 18–22 months of corrected [6]. Similarly, Rabe et al. [12] used the Bayley-III method to compare neurodevelopmental differences in cognitive, motor, and language development in 58 preterm infants who had either DCC or UCM at birth. The researchers examined 67% of the infants at 2 years of age and 50% at 3.5 years of age. They found that, there were no significant differences between the two groups in terms of developmental scores in the cognitive, language or motor domains [12]. Katheria et al. [13] assessed 74% of 197 infants of main study and found that Infants randomized to UCM at birth had significantly higher cognitive and language composite scores on the Bayley-III at 22–26 months corrected age and were less likely to have a cognitive composite score of < 85. The results of our study are consistent with the findings of Rabe et al. [12] and Katheria et al. [13], which demonstrated that UCM was not associated with adverse neurodevelopmental outcomes compared to DCC, and in some domains, such as cognitive and language development, may offer potential advantages.

Recently, El-Naggar et al. [11] reported that neurodevelopmental outcomes (Bayley-III cognitive, language, and motor scores) were similar at a corrected age of 36 months for very preterm infants randomized to the UCM or ICC groups at birth. Their follow-up compliance rate was 92%. El‑Naggar et al. [11] suggested that there were no RCT trials or systematic reviews that had reported an increased risk of severe IVH when UCM was compared to ICC in preterm infants [6, 11, 14, 18, 19]. Furthermore, another systematic review and meta-analysis reported that UCM was associated with lower odds of IVH compared to ICC [2]. In our main study, grade ≥ 3 IVH was detected in 3 preterms in the UCM group, but the difference between the groups was not statistically significant [14]. Two of these infants died during the neonatal period. The other infant with IVH who was planned for long-term neurodevelopmental evaluation could not be contacted. Consequently, there were no infants with grade ≥ 3 IVH in either the UCM or ICC groups in the present study. High-grade IVH is a well-established independent risk factor for adverse neurodevelopmental outcomes in very preterm infants [20]. In the present study, only one infant had IVH, and it was low grade. In addition, necrotising enterocolitis (NEC) has been associated with an increased risk of cognitive, motor, and language delays, particularly among preterm infants requiring surgery [21]. In the present study, only one infant had NEC which was not need to surgery. There was no statistically significant difference in the incidence of IVH or NEC between the study groups.

Although it was not the main objective of this study, we found that children’s neurodevelopmental status were associated with family income status, regardless of the method of placental transfusion. Children who grew up in households with incomes higher than the national minimum wage had better neurodevelopmental outcomes than those who grew up in households with incomes equal to or lower than the national minimum wage. The number of cases with family income equal to or lower than the minimum wage was not significantly different between the groups.

This study has several strengths, including a relatively high follow-up rate (77.5%) many years after the original trial, blinded outcome assessment to reduce detection bias, and the use of comprehensive and validated neurodevelopmental tools. However, it also has limitations. The findings’generalizability is limited by the small sample size, therefore they should be interpreted with caution. Larger, well-powered studies are needed to validate these findings. Furthermore, the lack of data on certain environmental variables (e.g., early intervention programs, home stimulation) prevents full adjustment for potential cofounders.

In conclusion, our findings indicate that I-UCM is not associated with long-term neurodevelopmental adverse effects in very preterm infants born at around 28 weeks of gestation without severe IVH. On the contrary, I-UCM may be linked to better cognitive and written language outcomes in early school-age years. Although these results support the continued consideration of I-UCM as a viable placental transfusion strategy when DCC is not feasible, larger studies are needed to confirm these potential benefits.

## Data Availability

The original data can be accessed via the following URL: https://tez.yok.gov.tr/UlusalTezMerkezi/tezSorguSonucYeni.jsp.

## Abbreviations

BAER: Brainstem auditory evoked response
BAYLEY-III: Bayley scales of infant and toddler development III
CS: Cesarean section
DCC: Deferred/delayed cord clamping
ICC: Immediate cord clamping
I-UCM: Intact- umbilical cord milking
IVH: Intraventricular hemorrhage
RCT: Randomized controlled trial
RBC: Red blood cell
UCM: Umbilical cord milking/stripping
Vineland-II: Vineland Adaptive Behavior Scales, Second Edition
WISC-IV: Wechsler Intelligence Scale for Children, Fourth Edition
