# Correction to: School age outcomes of very premature infants randomized to cord milking versus early cord clamping at birth

**DOI:** 10.1007/s00431-025-06390-4

**Published:** 2025-08-22

**Authors:** Kamile Akyol Ozkara, Serdar Alan, Ezgi Ozalp Akin, Emel Okulu, Emine Bahar Bingoler Pekcici, Elcin Caglar, Omer Erdeve, Begüm Atasay, Saadet Arsan

**Affiliations:** 1 Department of Pediatrics, Ankara University School of Medicine, Ankara, Turkey; 2 Division of Neonatology, Department of Pediatrics, Kirikkale University School of Medicine, Yahsihan/Kırıkkale, Turkey; 3 Division of Developmental Behavioral Pediatrics, Department of Pediatrics, Ankara University School of Medicine, Ankara, Turkey; 4 Division of Neonatology, Department of Pediatrics, Ankara University School of Medicine, Ankara, Turkey

**Correction to: European Journal of Pediatrics (2025) 184:462**

10.1007/s00431-025-06299-y

The authors of the published article titled “School age outcomes of very premature infants randomized to cord milking versus early cord clamping at birth” wish to correct an omission. Elcin Caglar, clinical psychologist, contributed significantly to this study. She was involved in the conception, ethics committee application, administration of the WISC-IV tests, and evaluation of the results. Her name should have been included as the sixth author.

The corrected author list is as follows: Kamile Akyol Ozkara, Serdar Alan, Ezgi Ozalp Akin, Emel Okulu, Emine Bahar Bingoler Pekcici, Elcin Caglar, Omer Erdeve, Begüm Atasay and Saadet Arsan.

The original article has been corrected.

## Data Availability

The original data can be accessed via the following URL: https://tez.yok.gov.tr/UlusalTezMerkezi/tezSorguSonucYeni.jsp.

